# Accurate Boron Determination in Tourmaline by Inductively Coupled Plasma Mass Spectrometry: An Insight into the Boron–Mannitol Complex-Based Wet Acid Digestion Method

**DOI:** 10.3390/molecules29112701

**Published:** 2024-06-06

**Authors:** Xijuan Tan, Yonggang Feng, Ruili Zhou, Denghong Wang, Ting Liang, Yan Wang

**Affiliations:** 1 Laboratory of Mineralization and Dynamics, College of Earth Sciences and Land Resources, Chang’an University, 126 Yanta Road, Xi’an 710054, China; ygfeng@chd.edu.cn (Y.F.); z199812068@163.com (R.Z.); liangt@chd.edu.cn (T.L.); 2 Xi’an Key Laboratory for Mineralization and Efficient Utilization of Critical Metals, Xi’an 710054, China; 3 Chinese Academy of Geological Sciences, Beijing 100037, China

**Keywords:** boron quantification, tourmaline, boron–mannitol complex, wet acid digestion, ICP-MS

## Abstract

Tourmaline, a boron-bearing mineral, has been extensively applied as a geothermometer, provenance indicator, and fluid-composition recorder in geological studies. In this paper, the decomposition capability of an HF-HNO_3_–mannitol mixture for a tourmaline sample was investigated in detail for the first time, and a wet acid digestion method based on the boron–mannitol complex for accurate boron determination in tourmaline by inductively coupled plasma mass spectrometry (ICP-MS) was proposed. With a digestion temperature of 140 °C, tourmaline samples of 25 mg (±0.5 mg) can be completely decomposed by a ternary mixture, which consisted of 0.6 mL of HF, 0.6 mL of HNO_3_, and 0.7 mL of 2% mannitol (wt.), via a continuous heating treatment of 36 h. Following gentle evaporation at 100 °C, the sample residues were re-dissolved using 2 mL of 40% HNO_3_ solution (wt.) and diluted to about 2.0 × 10^5^-fold by a two-step method using 2% HNO_3_ solution (wt.). The boron contents in a batch of parallel tourmaline samples were then determined by ICP-MS, and results showed that the boron concentration levels were in a range of 3.20–3.44% with determination RSDs less than 4.0% (*n* = 5). It was found that the boron concentrations obtained at the mass of ^10^B were comparable with results from the measurements at the mass of ^11^B. This revealed that the usage of 2% mannitol with a quantity as high as 0.7 mL in this developed approach did not exhibit significant effect on the quantification accuracy of boron at the mass of ^11^B. It was also found that the processes including fluoride-forming prevention and fluoride decomposition deteriorated the boron-reserving efficiency of mannitol for tourmaline, causing the averaged boron contents to vary from 2.25% to 3.57% (*n* = 5). Furthermore, the stability of the boron–mannitol complex under 185 °C by applying the laboratory high pressure-closed digestion method was evaluated, which showed that there existed a 60.36% loss of boron compared to that under 140 °C by using this proposed approach. For this ternary mixture, the tourmaline decomposing efficiency was found to be weakened prominently using 100 °C as the digestion temperature, and tourmaline powders can be observed even after 72 h of continuous heating with B contents within 1.09–1.23% (*n* = 5). To assess the accuracy of this developed method, the boron recovery of anhydrous lithium tetraborate was studied. It was found that the boron recoveries were within 96.59–102.12% (RSD < 1%, *n* = 5), demonstrating the accuracy and reliability of this proposed method, which exhibits advantages of high B preserving efficiency, and giving concentration information of both B and trace elements simultaneously. By applying such a boron–mannitol complex-based wet acid digestion method, the chemical composition of boron and trace elements in three tourmaline samples from different pegmatites were quantified, which provided valuable information to distinguish regional deposits and the associated evolution stages.

## 1. Introduction

Tourmaline is a borosilicate mineral with a general formula of XY_3_Z_6_(BO_3_)Si_6_O_18_(OH)_4_ [1,2], which can accommodate a wide range of different elements in trace levels [3]. This main boron (B)-bearing mineral usually occurs as dravite, buergerite, elbaite, schorl, uvite, etc. [4]. Being an accessory mineral, tourmaline can be found in hydrothermal ore deposits, and various rock types [5,6]. For example, tourmaline is an abundant phase in granites and associated hydrothermal rocks since its crystallization can occur from the early magmatic stage to the later sub-solidus fluids [7]. Such a mineral is chemically stable from conditions near the Earth’s surface to the pressures and temperatures of the upper mantle [8,9]. Furthermore, the extremely low rates of volume diffusion allow tourmaline to retain a chemical signature from the rock in which it develops [10]. This exceptional stability of tourmaline makes it a good recorder for the conditions in igneous, sedimentary, metamorphic, and hydrothermal settings. Accordingly, tourmaline has been extensively applied in geological studies as a geothermometer, provenance indicator, and fluid-composition recorder [6,11,12,13,14].

By analyzing the specific varieties of chemical compositions in tourmaline samples, Marks et al. [15] found that there were correlations of trace elements with major elements and potential crystal chemical effects on the incorporation of trace elements. The authors thus concluded that the trace element characteristics of tourmaline samples from pegmatitic and hydrothermal systems can allow us to distinguish fluid sources and unravel the chemical complexity of granite-related hydrothermal systems. Feng et al. [16] observed that the concentrations of elements Li, Mn, and Zn in black tourmalines from barren to Li pegmatites increased with the increasing differentiation degree of the pegmatites, while the values of δ^11^B showed an opposite trend. They therefore deduced that the geochemical characteristic of tourmaline can be used to indicate the mineralization of pegmatite-type Li deposits. Zhang et al. [17] identified the types of tourmaline samples from barren pegmatite and ore-bearing pegmatite via element and isotope composition studies, and then discussed the magmatic-hydrothermal evolution of West Kunlun orogen in China by using the chemical changes of tourmaline samples. Collectively, applying tourmaline as a geochemical tracer was generally based on its B isotope and element composition. However, the concentration information of B which is a major component in tourmaline was usually skipped. 

The element B, having two stable isotopes (i.e., ^10^B and ^11^B), belongs to the nonmetal lithophile elements and exhibits the property of moderate to high mobilization [18,19], and the B content in tourmaline is known to depend on the genesis process [20,21]. By taking the county of Cornubia in southwest England as an example, London and Manning investigated the relations of element B and tourmaline in magmatic-hydrothermal systems [22]. They showed that the magmas cannot conserve B in the form of tourmaline when there were low Fe-Mg contents, and indicated that most hydrothermal or metasomatic tourmaline lay in rocks that surround the magmatic sources of B. Furthermore, the authors proposed that the large quantities of tourmaline should be ascribed to the mixing of fluids containing B and Fe-Mg components, and thus concluded that the element B was an important constituent of the magmas in the studied area. Raith et al. [23] reported that the rare earth elements did not fractionate during tourmaline crystallization from boron-rich granitic melts or during alteration of clastic metasediments by boron-rich magmatic-hydrothermal fluids, but the B metasomatism can result in the systematic enrichment of B and the depletion of Li, K, Rb, Cs, and Mn in tourmaline rocks compared to unaltered metasedimentary host rocks in the Bonya Hills of Australia. It is no doubt that B concentration in tourmaline might highly correlate with the mineralization process, metasomatism, and metamorphism; therefore, it is necessary to incorporate B content details of tourmalines in the discussion of associated microenvironments of fluids, magmatic-hydrothermal fluid interactions, diagenesis, and mineralization.

It is known that tourmaline is characterized by a complex crystal structure, in which three B cations combine with O forming BO_3_ triangles. For each BO_3_ triangle, it shares one corner with the nine-coordinated polyhedron and the other two corners with the octahedra of (Al,Fe^3+^,Mg)-(O,OH) which connects with one Si-O tetrahedra through a shared O [24]. Previous studies show that the concentration of B in tourmaline can be determined by neutral-induced radiography [25], spectrophotometry [26], and inductively coupled plasma atomic/optical emission spectroscopy (ICP-ASE/OES) [27,28]. Among these methods, ICP-AES/OES was frequently used for B measurement in tourmaline. However, the release of B from the rhombohedral crystal of tourmaline is a prerequisite for ICP-AES analysis. Lihareva et al. [28] decomposed 0.2 g of the tourmaline sample by the fusion method using 1 g of the mixture of anhydrous Na_2_CO_3_ and ZnO with a ratio of 3.5:0.6. After sample dissolution using 30 mL of water and three drops of 96% ethanol on a steam bath, the B content in the tourmaline samples were determined by ICP-AES, and results showed that the B concentrations ranged from 2.11% to 3.10%, with relative standard deviations (RSDs) less than 5.2% (*n* = 4). Despite the high decomposition efficiency of tourmaline, the fusion method is usually notorious for introducing large quantities of the matrix due to flux addition [29], causing the accuracy deterioration of B quantification and B isotope analysis. 

Wet digestion with oxidizing acids, the most common sample preparation protocol, which exhibits the merits of being effective on both inorganic and organic materials, low matrix influence, and less interferences [30], is an alternative strategy to decompose tourmaline. Since Berzelius first made use of the conversion of SiO_2_ to SiF_4_ by using HF for analytical purposes in 1831 [31], HF has been a favorable reagent to decompose Si-O bonds in silicate samples [32]. However, with the usage of HF, the formation of BF_3_ which has a low boiling point (−100.3 °C, b.p.) can lead to B loss even at room temperature and result in a low recovery of B for samples [33]. Furthermore, B in acidic solutions is easily volatilized during the process of evaporation to dryness relative to a neutral or alkaline medium [34]. Chen et al. [35] studied the effect of H_3_PO_4_ on B reservation in tourmaline digestion by HF-HNO_3_-HClO_4_, showing that the contents of B_2_O_3_ from the ICP-OES analysis were 6.90–10.10%. But the presence of P and Cl causes interferences in the quantification of trace elements, such as Cr, Cu, Zn, and Ga [36]. Furthermore, the stable complex between H_3_PO_4_ and B makes subsequent B isotope analyses challenging. By using mannitol (C_6_H_14_O_6_) as the B preserving reagent, Liu et al. [37] proposed an ultrasonication-assisted low-temperature digestion method for silicate samples via the HF-HNO_3_ mixture at 65 °C, yielding B recoveries for the silicate standard material JB-2a higher than 75%. With the ternary mixture of HF-HNO_3_–mannitol as the digestion reagent, our previous work showed that the B recoveries for silicate standard materials W-2, JB-2a, and JR-2 were within 95.5–105.5% under a digestion temperature between 65 °C and 140 °C, and the ^10^B in ICP mass spectrometry (ICP-MS) was preferable to ^11^B when samples contained B over 7250 ng in particular [38]. However, to the best of our knowledge, there has been no study on the B recovery of this HF-HNO_3_–mannitol mixture for tourmaline up to date. 

In this current work, the analytical efficiency of the HF-HNO_3_–mannitol mixture-based wet method for the B quantification of tourmaline was investigated for the first time. The determination properties of ^10^B and ^11^B in ICP-MS, the step of fluoride prevention and decomposition, and the stability of the B–mannitol complex were discussed in detail. Furthermore, the accuracy of this developed method was evaluated by a B recovery study of anhydrous lithium tetraborate (Li_2_B_4_O_7_). Finally, this proposed wet acid digestion method was applied to B concentration and trace element quantification by ICP-MS for a series of tourmaline samples.

## 2. Results and Discussion

### 2.1. Digestion Method Based on HF-HNO_3_–Mannitol for Tourmaline Sample

It is known that the tourmaline structure is characterized by ditrigonal rings of six tetrahedra, and this structure complexity leads to the great compositional flexibility of tourmaline samples. Such a complex structure and composition can cause difficulties in tourmaline decomposition [39]. Considering the digestion property of the HF-HNO_3_ mixture for silicate samples [32,40] and the B reservation of mannitol [41,42], the analytical efficiency of the ternary mixture of HF-HNO_3_–mannitol for tourmaline was investigated in detail.

#### 2.1.1. Digestion Temperature and Measurement Properties of ^10^B and ^11^B

In our previous work, it was found that there was no obvious effect of the digestion temperature in a range of 65–140 °C on the B recoveries of silicates by using a wet acid method based on the ternary mixture of HF-HNO_3_–mannitol [38]. By taking the structure complexity of tourmaline and time consumption into consideration, the temperature of 140 °C was chosen as the decomposition temperature for tourmaline samples in this current work. Following the digestion method M_Tour_-1, clear solutions for tourmaline NYS2 with 26 parallel samples were found to be obtained after 36 h of continuous heating. The B contents in the final solutions of this sample batch were quantified by ICP-MS at the masses of both ^10^B and ^11^B, and the determination results were summarized in Table 1 with RSDs less than 3.45% (*n* = 5).

As shown in Table 1, the B contents (wt. %) for this studied tourmaline NYS2 varied from 2.25% to 3.57%. Such a prominent variation revealed that there existed a loss of B during the sample digestion process, and the potential reason resulting in the changeable preserving efficiency of mannitol for element B is discussed in the following chapter. It can also be seen from Table 1 that there was no significant difference in the B concentrations between measurements at the mass of ^11^B and at the mass of ^10^B. Despite the addition of 0.7 mL of 2% mannitol due to the fact that the B content in 25 mg of tourmaline might be around 875 μg, it is worth noting that the ^12^C interference from the large quantity of mannitol on the ^11^B measurement was not distinctive. This result contrasted with the previously observed phenomenon that the accuracy of B quantification at the mass of ^11^B deteriorated when B content was higher than 7250 ng [38]. Thus, the comparable results from measurements at the masses of ^11^B and ^10^B might be ascribed to the high dilution factor of about 2.0 × 10^5^. Apparently, both the masses of ^11^B and ^10^B in ICP-MS are plausible in the B measurement of tourmaline samples by using the proposed digestion approach.

The digestion efficiency of this ternary mixture for tourmaline with 100 °C as the decomposition temperature was also evaluated. It was found that there were certain amounts of tourmaline NYS2 powders observed by the naked eye even after 72 h of continuous heating. The ICP-MS quantification results showed that the B contents were within 1.09–1.23% (*n* = 5). This revealed that the lower temperature relative to 140 °C weakened the decomposing ability of the HF-HNO_3_–mannitol mixture for tourmaline, which reconfirmed the structure complexity of tourmaline. Collectively, 140 °C was favored as the decomposition temperature for tourmaline samples.

#### 2.1.2. Effect of Insoluble Fluorides on B Quantification of Tourmaline

With M_Tour_-1 as the sample digestion method, it is noteworthy that there was a significant variation in B content for the same tourmaline sample. For example, the obtained B concentrations from measurements at the mass of ^10^B were between 2.25% and 3.31% (wt. %), giving an average value of 2.98 ± 0.08% (2σ, *n* = 26). Despite this yielded average value falling within the reported B concentration range of tourmaline [43], the deviation between the highest and lowest concentrations reached 1.06%. Since the molar ratio of the added mannitol to B in tourmaline is 0.996 (one should note this value is from the ideally highest B content of 3.5%), which is higher than the required mannitol/B molar ratio of 0.781 [44], the observed remarkable variation should not be due to the quantity of mannitol. Hence, it can be deduced that the scattering results of B content might come from the processes of fluoride-forming prevention and fluoride decomposition. 

To further assess the influence of insoluble fluorides on the quantification accuracy of B in tourmaline, the digestion method M_Tour_-2 which skipped the addition of 8% HNO_3_ solution and 6% HCl solution was studied with the results collected in Table 1. The B contents for another batch of 10 parallel samples of tourmaline NYS2 from the measurement at the mass of ^10^B were found to be within 3.20–3.52%, yielding an average value of 3.33 ± 0.06% (2σ, *n* = 10). Apparently, the deviation between the highest and lowest concentrations declined to 0.32%. Thus, there is no necessity to deal with the insoluble fluorides formed during tourmaline digestion, which improves the precision of B analysis and simplifies the whole digestion process with less time consumption. However, currently, the relationship between the repetition of the digestion method and the fluoride handling procedure remains unclear. 

#### 2.1.3. Stability of B–Mannitol Complex under High Temperature

In this work, the stability of the complex between B and mannitol was evaluated by comparing the B results from M_Tour_-2 with a digestion temperature of 140 °C and M_Tour_-3 which utilized a digestion temperature of 185 °C. Here, the temperature of 185 °C is a default value in rock sample digestion using the laboratory high-pressure-closed wet acid method, which is efficient for the complete decomposition of various types of rocks [40]. As can be seen in Table 1, the B contents from measurements at the masses of ^10^B and ^11^B were 1.20–1.56% and 1.20–1.57%, respectively. The corresponding average values of B contents were 1.29 ± 0.06% and 1.30 ± 0.08% (2σ, *n* = 10). It is also clear that the results from measurements at the mass of ^10^B did not show differentiation from those from measurements at the mass of ^11^B, which reconfirmed that both ^10^B and ^11^B can be equally applied to the B quantification of tourmaline samples. The further comparison of the average B contents showed that there existed a decrement of 60.36% when using the digestion method M_Tour_-3, revealing that the B–mannitol complex cannot stabilize at 185 °C. Hence, the digestion method M_Tour_-2 in which the decomposition temperature was set as 140 °C was preferable for tourmaline samples. 

### 2.2. Accuracy of the Proposed HF-HNO_3_–Mannitol-Based Digestion Method

It is known that the accuracy of an analytical method is typically assessed by using standard reference material. Since the standard reference material of tourmaline was not available in this current work, the accuracy of the proposed HF-HNO_3_–mannitol-based digestion method M_Tour_-2 was then evaluated by a B recovery study of anhydrous Li_2_B_4_O_7_, which provides borate species [44] to form boron–mannitol chelate complexes [45]. With 10 mg of this reagent treated by method M_LT_-1 in which the quantity of 2% mannitol was increased to 2.5 mL according to sample B content [46], the B concentration was determined with the results collected in Table 2. Clearly, the obtained B contents of anhydrous Li_2_B_4_O_7_ (2σ, *n* = 5) were in the range of 25.28 ± 0.27% for measurement at the mass of ^10^B and 25.85 ± 0.31% for the measurement at the mass of ^11^B, yielding the corresponding B recoveries within 96.59–99.35% and 98.70–101.76%. Furthermore, the reliability of this assessment strategy via anhydrous Li_2_B_4_O_7_ for the developed method was tested by the method M_LT_-2, which only used the binary mixture of HNO_3_–mannitol to exclude the potential B loss from the concentrated HF. As shown in Table 3, the B recoveries from method M_LT_-2 were within 96.96–102.12% (2σ, *n* = 5), which were consistent with those from method M_LT_-1. This demonstrated that the reagent mannitol can avoid B loss under acidic environments with the existence of HNO_3_ during the evaporation process and efficiently exclude the formation of volatile BF_3_ from the usage of HF. It can thus be concluded that the accuracy assessing strategy through anhydrous Li_2_B_4_O_7_ analysis for the proposed digestion method M_Tour_-2 was reasonable. Hence, the digestion method M_Tour_-2 based on the HF-HNO_3_–mannitol mixture is reliable and robust for accurate B quantification of tourmaline samples.

### 2.3. Application of Proposed Method for B and Trace Element Quantification in Tourmaline

In this work, the B contents in three tourmaline samples with different colors (i.e., pink tourmaline NYS2, black tourmaline NYS8-4, and green tourmaline XHST) were determined by using the proposed M_Tour_-2 as the sample digestion method. The results in Table 3 showed that the B contents (2σ, *n* = 5) were 3.33 ± 0.06%, 2.79 ± 0.03%, and 3.04 ± 0.21% for NYS2, NYS8-4, and XHST, respectively. This prominent difference in B contents revealed the complexity of the studied tourmaline samples. Thus, the trace elements in these three tourmaline samples were then investigated (see Table 3). Results showed that the trace elements of the studied tourmaline samples also exerted characteristic patterns. In general, Li concentration increased with increasing B content, while element Zn decreased with increasing B content. It was found that the pink tourmaline NYS2 had relatively high contents of Ga, Rb, and Cs, while the black tourmaline NYS8-4 was characterized by the enrichment of elements Be, Zn, Ga, Zr, Nb, Hf, Ta and U. It is interesting that the green tourmaline XHST, from Xiaohusite pegmatite in Xinjiang of China, was observed to enrich with elements Pb and Bi, with the concentration of Bi specifically as high as 333.26 ± 4.61 μg/g. Since the tourmaline samples NYS2 and NYS8-4 were from the granitic pegmatites in East Qinling of China [16], the concentration patterns of B and trace elements might result from the differentiation degree of the pegmatites. Hence, the geochemical characteristic of tourmaline can allow for region distinguishing and provide valuable information to investigate the associated evolution stages of pegmatites.

## 3. Materials and Methods

### 3.1. Chemical Reagents and Instrumental Apparatus

The guaranteed grade reagent mannitol and acids including HNO_3_ (68% *v*/*v*, AR grade), HF (40% *v*/*v*, AR grade), and HCl (36% *v*/*v*, AR grade) were from the China National Pharmaceutical Group Co. Ltd. In this work, all the acids were purified twice using sub-boiling distillation in Teflon stills (Savillex DST-1000-PFA, Eden Prairie, MN, USA) prior to usage. The AR-grade anhydrous Li_2_B_4_O_7_ was purchased from Luoyang Chaonai Experimental Equipment Co. LTD (Luoyang, Henan, China). The single-element standard solution Rh (1.0 mg/mL) and B (1.0 mg/mL), and the multi-element standard solution (100 μg/mL) were purchased from the National Institute of Standards and Technology, China. Ultrapure water with a resistivity of 18.2 MΩ·cm was obtained by a Milli-Q^®^ EQ 7000 water purification system (Millipore, Bedford, MA, USA). 

An Agilent 7900 ICP-MS (Agilent, Santa Clara, CA, USA) was utilized to carry out B determination. This ICP-MS instrument contained a peristaltic pump, a concentric quartz nebulizer, a Scott-type double spray chamber, a standard quartz torch, an assemblage of Ni sample/skimmer cones (1.0/0.45 mm), a quadrupole mass analyzer, and an orthogonal detector system. A Peltier-cooling system was applied to control the spray temperature in a range of −5–20 °C, and a Pt shielding plate and a silicon shielding cap were equipped to enhance the element signal sensitivity of this ICP-MS.

### 3.2. Reagents Preparation

In this work, the preparation of solutions was completed through a gravimetric protocol. By using ultrapure water, the 8% HNO_3_ (wt.) solution and 6% HCl solution (wt.) were prepared by diluting concentrated HNO_3_ of 0.4 mL and concentrated HCl of 0.3 mL to 50 g, respectively. The 2% mannitol solution (wt.) was prepared by dissolving the mannitol of 1.6 g using ultrapure water with a final solution weight of 80 g. The B external calibrators with concentrations of 5, 25, 50, 100, and 150 ng/mL were prepared progressively from the single-element standard solution B by using a 2% HNO_3_ solution (*v*/*v*). Here, a solution of 2% HNO_3_ (*v*/*v*) without B addition was utilized as the blank external calibrator. Here, to exclude any possible assay bias from long-term storage, all standard solutions were prepared freshly. The Rh solution of 500 ng/g as the online internal standard was prepared by diluting 50 μL of 1.0 mg to 100 g using a 2% HNO_3_ solution (*v*/*v*).

### 3.3. Sample Handling Description

The sample preparation was carried out in a one hundred cleanroom. The associated evaporation and heating processes were performed in a B-free ULPA filtration hood. The PFA beakers and PTFE bombs utilized as sample digestion containers were immersed in an aqua regia solution (HNO_3_-HCl, 1:3, *v*/*v*) and heated on a hotplate at 120 °C for 24 h, followed by ultrapure water treatment under the same condition. These containers were carefully rinsed three times with ultrapure water and let dry before usage. 

The tourmaline samples were digested by three methods based on the HF-HNO_3_–mannitol mixture according to the following procedures: (1) Samples with a quantity of 25 mg (±0.5 mg) were weighed in 15 mL of PFA beakers (M_Tour_-1, M_Tour_-2) or PTFE bombs (M_Tour_-3). (2) 0.6 mL of concentrated HF, 0.6 mL of concentrated HNO_3,_ and 0.7 mL of 2% mannitol were added gently into sample containers. (3) When the reagents mixed well with samples, the sample containers were sealed tightly, and then placed on a hotplate at 140 °C for 36 h (M_Tour_-1, M_Tour_-2) or put in an oven with a temperature of 185 °C for 24 h (M_Tour_-3) [40]. (4) Thereafter, sample solutions were evaporated to incipient dryness at 100 °C. Here, only for method M_Tour_-1, 0.6 mL of 8% HNO_3_ solution was added into the incipient dry samples and fluxed overnight. The samples were evaporated to incipient dryness again and fluxed overnight in 0.6 mL of a 6% HCl solution. After the second flux, the samples were heated until incipient dryness was obtained. (5) The incipiently dry samples were then fluxed in 2.0 mL of 40% HNO_3_ solution (*v*/*v*) for 4 h at 100 °C. (6) The solutions were then transferred to PET bottles after aging overnight and gravimetrically diluted to about 50 g using 2% HNO_3_ (*v*/*v*), from which 0.5 mL of aliquot was transferred to another PET bottle and then diluted 100-fold to obtain the final sample solution for B quantification by ICP-MS.

In the method evaluation study, 10.00 ± 0.50 mg of Li_2_B_4_O_7_ were weighted in 15 mL of PFA beakers, with a ternary mixture (i.e., 0.6 mL of concentrated HF, 0.6 mL of concentrated HNO_3_, and 2.5 mL of 2% mannitol) added (M_LT_-1) or binary mixture (i.e., 0.6 mL of concentrated HNO_3_ and 2.5 mL of 2% mannitol) added (M_LT_-2). The sample containers were then capped tightly and heated on a hotplate at 140 °C for 36 h. Thereafter, sample solutions were evaporated to incipient dryness at 100 °C and added with 2.0 mL of 40% HNO_3_ solution (*v*/*v*). After being fluxed for 4 h and aging overnight, the samples were gravimetrically diluted to 50 ± 0.5 g using 2% HNO_3_ (*v*/*v*), from which 0.1 mL of aliquot was diluted to about 50 g. This final sample solution with a total dilution factor of around 2.5×10^6^ was taken for B quantification by ICP-MS directly.

### 3.4. Operating Conditions of ICP-MS

After the ICP-MS instrument had been warmed up for at least one hour, the daily optimization to obtain the highest possible sensitivities for low- to high-mass isotopes was carried out. All the details were given in our previous work [38]. In brief, a tuning solution that contained 1.0 ng/mL of elements Li, Y, Ce, and U was introduced into the ICP-MS system, and the position of the torch axis, EM value, lens voltages, mass resolution, and mass axis were adjusted automatically, with oxide formation (CeO^+^/Ce^+^) and doubly charged species (Ce^2+^/Ce^+^) controlled lower than 2.0%. Under the optimized operating conditions, the detector was then calibrated using a 50 ng/mL multi-element solution to achieve desirable P/A factors for pulse and analog modes. Before any quantification, the final solution of one digested tourmaline sample uptake was used to flush the ICP-MS system for over 30 min. Here, a standard solution of 50 ng/mL as the instrumental drift monitor was measured after the analysis of every five unknown samples. Furthermore, the whole system was washed with 2% HNO_3_ solution (*v*/*v*) after every quantification to minimize the memory effect. In this work, no gas mode of this ICP-MS and Rh solution as an internal standard was applied to B determination. The typical operating conditions of this ICP-MS configuration for B measurement are summarized in Table 4.

## 4. Conclusions

This work proposed a robust HF-HNO_3_–mannitol mixture-based wet digestion method for accurate B quantification in tourmaline by ICP-MS. After continuous heating at 140 °C for 36 h, 25 mg (±0.5 mg) of the tourmaline sample can be completely decomposed by the mixture of 0.6 mL of HF, 0.6 mL of HNO_3,_ and 0.7 mL of 2% mannitol. The results showed that there was no difference in B measurement at the masses of ^10^B and ^11^B, revealing that this developed method with usage of 2% mannitol as high as 0.7 mL did not affect B quantification accuracy at the mass of ^11^B. It was also found that skipping the procedures of fluoride-forming prevention and fluoride decomposition can improve the B-retaining efficiency of mannitol for tourmaline, and the corresponding deviation between the highest and lowest B concentrations declined from 1.06% to 0.32%.

The digestion efficiency evaluation of the HF-HNO_3_–mannitol mixture revealed that the lower temperature (i.e., 100 °C) can weaken the decomposing ability of this ternary mixture for tourmaline. Furthermore, the stability study of the B–mannitol complex showed that the B loss reached 60.36% under the decomposition temperature of 185 °C compared to that under 140 °C. It can thus be deduced that the temperature of 140 °C is optimal for tourmaline decomposition. With recoveries ranging from 96.59% to 102.12% (RSD < 1%, *n* = 5), the B recovery study of anhydrous Li_2_B_4_O_7_ demonstrated the accuracy and reliability of this wet digestion method based on the ternary mixture of HF-HNO_3_–mannitol.

By applying this proposed HF-HNO_3_–mannitol mixture-based wet digestion method, the concentrations of B and trace elements in three tourmaline samples from different pegmatite deposits were accurately quantified. Results showed that the B content was highly correlated with trace element compositions. With the obtained concentration patterns of B and trace elements, the regional deposits were successfully distinguished, and the information on the evolution stages of pegmatites was also identified accordingly. Collectively, the geochemical characteristics of both B and trace elements in tourmaline can be simultaneously obtained by using this developed approach, which is of great significance to have the full information of tourmaline samples, promising the application of tourmaline in associated geological process investigations. Additionally, this proposed wet digestion method based on the ternary mixture of HF-HNO_3_–mannitol also sheds light on the B and trace element analysis in other B-bearing minerals, such as datolite, danburite, etc.

## Figures and Tables

**Table 1:** Results of B contents in tourmaline using different digestion methods ^1^.

Method	Sample	^10^B		^11^B		Method	Sample	^10^B		^11^B	
		Content	RSD%	Content	RSD%			Content	RSD%	Content	RSD%
M_Tour_-1	NYS2-1	3.09	0.69	3.13	0.65	M_Tour_-2	NYS2-27	3.38	0.57	3.40	0.75
	NYS2-2	3.01	0.34	3.04	1.18		NYS2-28	3.32	0.28	3.35	0.39
	NYS2-3	2.94	0.74	2.97	0.22		NYS2-29	3.52	0.73	3.55	0.55
	NYS2-4	2.92	1.32	2.96	1.38		NYS2-30	3.20	0.69	3.21	1.07
	NYS2-5	3.20	0.76	3.27	0.95		NYS2-31	3.44	2.44	3.43	2.81
	NYS2-6	3.22	0.36	3.50	0.61		NYS2-32	3.29	1.14	3.29	0.77
	NYS2-7	2.81	0.73	3.04	0.76		NYS2-33	3.26	1.10	3.31	0.78
	NYS2-8	3.24	0.17	3.51	0.25		NYS2-34	3.32	0.60	3.36	0.50
	NYS2-9	2.85	0.72	3.09	0.59		NYS2-35	3.21	1.01	3.18	0.78
	NYS2-10	2.84	0.51	3.09	0.63		NYS2-36	3.33	1.04	3.34	0.93
	NYS2-11	2.97	0.68	3.22	0.26		Min	3.20		3.18	
	NYS2-12	2.95	0.53	3.20	0.47		Max	3.52		3.55	
	NYS2-13	2.99	0.72	3.25	0.44		Average	3.33		3.34	
	NYS2-14	3.00	0.51	3.26	0.61		2σ	0.06		0.07	
	NYS2-15	3.31	1.02	3.57	0.72	Method	Sample	^10^B		^11^B	
	NYS2-16	3.07	0.45	3.32	0.78			Content	RSD%	Content	RSD%
	NYS2-17	3.10	0.71	3.34	0.31	M_Tour_-3	NYS2-37	1.20	0.63	1.20	1.54
	NYS2-18	2.96	0.73	3.22	0.80		NYS2-38	1.26	0.67	1.27	0.83
	NYS2-19	3.09	0.77	3.34	0.34		NYS2-39	1.22	0.83	1.24	0.74
	NYS2-20	3.07	0.68	3.34	0.58		NYS2-40	1.28	1.04	1.29	0.77
	NYS2-21	3.03	0.72	3.04	1.09		NYS2-41	1.22	0.31	1.23	0.79
	NYS2-22	2.99	0.52	2.99	0.46		NYS2-42	1.46	1.12	1.48	1.61
	NYS2-23	2.90	3.47	2.91	3.15		NYS2-43	1.21	0.92	1.23	1.28
	NYS2-24	2.55	0.45	2.58	0.93		NYS2-44	1.56	0.78	1.57	1.14
	NYS2-25	2.25	1.30	2.27	1.02		NYS2-45	1.23	0.92	1.24	0.86
	NYS2-26	3.16	0.86	3.18	1.10		NYS2-46	1.23	0.42	1.24	1.11
	Min	2.25		2.27			Min	1.20		1.20	
	Max	3.31		3.57			Max	1.56		1.57	
	Average	2.98		3.14			Average	1.29		1.30	
	2σ	0.08		0.11			2σ	0.08		0.08	

^1^ B content is expressed in wt. % and the results are given in 95% confidential intervals (*n* ≥ 10).

**Table 2:** Boron contents in 10 mg of Li_2_B_4_O_7_ reagent from ICP-MS analysis ^1^.

Method	Sample	^10^B			^11^B		
		Content	RSD%	Recovery	Content	RSD%	Recovery
M_LT_-1	Li_2_B_4_O_7_-1	25.00	0.57	96.59	25.59	0.68	98.86
	Li_2_B_4_O_7_-2	25.04	0.57	96.76	25.55	0.64	98.70
	Li_2_B_4_O_7_-3	25.20	0.31	97.36	25.69	0.57	99.27
	Li_2_B_4_O_7_-4	25.43	0.39	98.26	26.09	0.37	100.82
	Li_2_B_4_O_7_-5	25.71	0.89	99.35	26.34	0.75	101.76
	Min	25.00		96.59	25.55		98.70
	Max	25.71		99.35	26.34		101.76
	Average	25.28		97.66	25.85		99.88
	2σ	0.27		1.03	0.31		1.20
M_LT_-2	Li_2_B_4_O_7_-6	25.30	0.26	97.74	26.00	0.29	100.47
	Li_2_B_4_O_7_-7	25.36	0.31	97.99	25.95	0.59	100.25
	Li_2_B_4_O_7_-8	25.42	0.52	98.20	26.01	0.95	100.49
	Li_2_B_4_O_7_-9	25.83	0.71	99.80	26.43	0.67	102.12
	Li_2_B_4_O_7_-10	25.09	0.60	96.96	25.78	0.27	99.62
	Min	25.09		96.96	25.78		99.62
	Max	25.83		99.80	26.43		102.12
	Average	25.40		98.14	26.03		100.59
	2σ	0.24		0.93	0.21		0.83

^1^ B content is expressed in wt. % and the results are given in 95% confidential intervals (*n* = 5).

**Table 3:** Content results of boron and trace elements in different tourmaline samples ^1^.

Element	NYS2		NYS8-4		XHST	
	Content	2σ	Content	2σ	Content	2σ
B	3.33%	0.06%	2.79%	0.03%	3.04%	0.21%
Li	9107	193	2802	42	6753	199
Be	14.35	0.28	96.32	0.64	9.81	0.48
Sc	0.16	0.01	0.31	0.001	0.43	0.004
V	0.03	0.01	0.34	0.01	14.93	0.01
Cr	0.30	0.11	0.10	0.004	0.59	0.02
Co	0.005	0.001	1.37	0.004	1.21	0.02
Ni	0.54	0.12	0.42	0.03	0.58	0.06
Cu	1.29	0.15	0.53	0.02	7.23	0.11
Zn	3.45	0.17	3360	11	264	2.38
Ga	62.85	3.55	40.14	0.52	41.39	0.03
Rb	102.3	1.60	3.14	0.10	2.39	0.13
Sr	19.78	0.26	8.21	0.11	26.26	0.14
Zr	0.24	0.03	10.44	4.07	0.22	0.00
Nb	7.52	0.07	115	25.78	4	0.09
Cd	0.54	0.01	0.15	0.001	0.18	0.003
Cs	34.69	0.60	8.08	0.30	1.97	0.07
Ba	0.12	0.03	0.44	0.09	0.31	0.12
Hf	0.13	0.012	2.33	0.73	0.03	0.00
Ta	10.97	0.37	293.0	64.2	1.7	0.17
Pb	105.38	1.08	5.35	0.002	126.39	2.27
Bi	13.38	0.11	0.57	0.005	333.26	4.61
Th	0.24	0.01	1.10	0.18	0.06	0.002
U	0.018	0.004	16.61	6.22	0.08	0.01

^1^ Element B is given in wt. % and trace elements are given in μg/g.

**Table 4:** Operating conditions of utilized ICP-MS for B determination ^1^.

Instrumental Parameter	Operating Condition
RF power	1550 W
Temperature of spray chamber	2 °C
Plasma gas (Ar)	15 L/min
Auxiliary gas (Ar)	1.0 L/min
Nebulizer gas (Ar) *	1.05 L/min
Sampling depth *	9.0 mm
Dwell time	300 ms
Settling time	0.2 ms
Collision and reaction cell mode	No gas
Detector mode	Dual (pulse and analog double mode)
Data-collecting mode	Peak jumping/hopping

^1^ The parameters with a star mark (*) are default values, which can be adjusted during optimization.

## Data Availability

Data are contained within the article.
